# Feasibility of HABIT-ILE@home in children with cerebral palsy and adults with chronic stroke: A pilot study

**DOI:** 10.1371/journal.pdig.0000850

**Published:** 2025-05-08

**Authors:** Edouard Ducoffre, Carlyne Arnould, Merlin Somville, Zélie Rosselli, Geoffroy Saussez, Yannick Bleyenheuft

**Affiliations:** 1 Motor Skill Learning and Intensive Neurorehabilitation Lab, Institute of neuroscience, UCLouvain, Louvain-la-Neuve, Belgium; 2 Forme & fonctionnement Humain (FfH) Lab, CeREF-Santé, Haute Ecole Louvain en Hainaut, Montignies-sur-Sambre, Belgium; Iran University of Medical Sciences, IRAN, ISLAMIC REPUBLIC OF

## Abstract

**Introduction:**

Children with cerebral palsy (CP) and adults with chronic stroke (CS) usually have disabilities in voluntary motor control. Hand-Arm Bimanual Intensive Therapy Including Lower Extremities (HABIT-ILE), an evidence-based therapy, has always been provided during day camps. This pilot study investigates if HABIT-ILE@home, a remote neurorehabilitation, is feasible for children with CP and adults with CS.

**Methods:**

Four children with CP (5-18y) and three adults with CS were recruited. They received 15h (5x3h) of HABIT-ILE@home provided by a caregiver with a remote supervision of 30min at the beginning and end of each session. A large touch screen, the REAtouch Lite, was used as a support for the therapy. An interview based on a questionnaire (n = 73 items for CP/ n = 74 items for stroke patients; scored from 0 “disagree” to 3 “agree”, a higher rating meaning a more positive aspect of the therapy) was conducted with patients and their caregivers after 15h of supervised home-therapy to assess their adherence to the treatment and the feasibility of HABIT-ILE@home. Performance and satisfaction in achieving functional goals were assessed before and after the intervention using the Canadian Occupational Performance Measure (COPM).

**Results:**

Caregivers felt sufficiently supported by the supervision team (medians = 3) to carry out HABIT-ILE@home sessions thanks to an adequate clinical supervision (CP median = 2.6; CS median = 2.9). HABIT-ILE principles were transferable at patients’ home (CP median = 2.6; CS median = 2.8). The impact of the therapy on daily organization was more problematic for children’s caregivers (median = 1.5) than for adults’ caregivers (median = 3). Children with CP enjoyed the therapy (median = 2) but felt that it was too long (median = 1) and significant fatigue was present (median = 1.3). CS adults did not find the therapy fun (median = 1) but considered it as extremely useful (median = 3). Although the motivational source differed between children and adults, this did not seem to strongly affect adherence to treatment. Performance and satisfaction in achieving functional goals improved over the MCID (2 points) for all CS participants and for 3 out 4 CP children.

**Conclusion:**

HABIT-ILE@home seems to be feasible for children with CP and adults with CS. It may allow more patients to benefit from an efficient neurorehabilitation, whatever sanitary conditions or patients’ home geographical locations.

## Author summary

People with neurological conditions such as chronic stroke or cerebral palsy often experience problems with voluntary motor control and difficulties in performing daily activities. Growing evidence suggests that goal-oriented intensive neurorehabilitation programs should be recommended as a first-line treatment (e.g., HABIT-ILE). Despite the excellent results in improving motor abilities and activities, they are still not widely adopted in clinical practice. The primary barriers include limited accessibility (due to financial/reimbursement issues and remote locations) and the scarcity of trained therapists. In this study, we aimed to investigate the feasibility of implementing HABIT-ILE in a home setting with caregivers and a remote supervision. Using a telerehabilitation device, caregivers provided 15 hours of therapy, including 5 hours of supervised sessions. Questionnaires were specifically developed to assess different aspects of this new modality of the therapy (HABIT-ILE@home), including the use of the device and the adherence of both patients and caregivers to the program. The results demonstrate that this therapy modality is feasible but also highlight significant differences between adults with chronic stroke and children with cerebral palsy, as well as their respective caregivers. These factors will need to be considered in future studies aiming at investigating the efficacy of this adaptation of HABIT-ILE.

## Introduction

Cerebral palsy (CP) and stroke are leading causes of disability for children [1,2] and adults [3], respectively. The prevalence for CP is around 2.11 per 1000 living birth [4] and stroke occurs in 1.322 out of 100 persons [5]. The consequences of brain lesions are highly variable depending on the timing, extent, and location of the lesions and the subsequent cortical reorganization. However, they usually include motor disorders (walking and grasping difficulties) leading to long-term functional limitations in daily activities and restrictions in life situations [1,6].

Conventional neurorehabilitation therapies for patients with brain lesions are historically based on neurodevelopmental techniques, which can usually include stretching to prevent secondary contractures and deformities and guided movements to recover normal movement patterns, among other techniques, with an active therapy time that may be low [7–9]. However, neurorehabilitation is in constant evolution and there is growing evidence that more recent interventions based on motor skill learning principles, may be more efficient in improving patients’ motor abilities and independence in daily life activities [10,11]. Constraint-Induced Movement Therapy (CIMT) is an intensive, goal-oriented therapy developed both for stroke adults [12] and children with CP [13] showing progress in hand functions and achievement of daily activities [14,15]. Other intensive therapies based on motor skill learning principles have been developed such as Hand-Arm Bimanual Intensive Therapy (HABIT) [16] or HABIT-Including Lower Extremities (HABIT-ILE) [17] to suit the bimanual need in daily activities. Both consist in intensive bimanual interventions, but HABIT-ILE also incorporates continuous postural control and lower extremities (LE) solicitations [18] with demonstrated improvements in the upper and lower extremities in unilateral [19] and bilateral CP [20,21].

Although HABIT-ILE is currently under investigation for adults with stroke [22], several evidence suggest that such a therapy might be efficient for this population as well [23]. First, motor improvements have already been observed in stroke patients using CIMT [11] sharing the same therapeutic motor skill learning principles as HABIT-ILE. Second, even considering specificities of mature or developing brain, both stroke and CP are characterized by the same type of brain lesions involving the corticospinal tract responsible for voluntary movements. Third, neuroplastic changes were observed both in adults with stroke [24,25] and children with CP [26,27] after motor skill learning interventions, suggesting that such interventions can be considered as a “windows of opportunity” where motor and daily activity performance can be improved in the long-term [28].

Up to now, HABIT-ILE has always been provided face-to-face by at least one therapist for each patient during intensive day camps [17]. Despite strong evidence of its effectiveness, it is not widely applied in clinical routine, notably due to limited number of therapists currently trained to apply this therapy and its limited accessibility (financial/reimbursement issues, remote areas). Patients living within rural settings may be less likely to have access to an optimal neurorehabilitation [29–31], especially intensive interventions. Home-based telerehabilitation might be a good way to reduce those inequalities [32]. In addition, pandemics such as COVID-19 have crystallized this issue as health care institutions may be severely disrupted in those circumstances; home-based telerehabilitation being almost the only solution for providing healthcare [33]. From this perspective, the World Stroke Organization (WSO) highlights the importance of using telerehabilitation programs and recommends the possibility to train patients’ caregivers, so they can deliver a therapy at home when there is limited access to care [34]. In addition, recent clinical practice guidelines for improving physical functions in children with CP recommend parental engagement for the success of any intervention [35]. Caregivers/relatives’ involvement in rehabilitation with rigorous coaching and support to the therapeutic plan could address the scarcity of trained therapists for such intensive programs and the accessibility issues.

Based on these recommendations, this pilot study will investigate the feasibility to implement a HABIT-ILE intervention at patients’ home, performed by a caregiver with a remote therapeutic supervision supported by a telerehabilitation device.

## Materials and methods

This is a prospective pilot study with a pre-post intervention design where family/relatives acted as caregivers. It was conducted by the Motor Skill Learning and Intensive Neurorehabilitation (MSL-IN) lab, UCLouvain in Brussels, Belgium, under the agreement (B403201316810) of the ethical committee *Hospitalo-Facultaire* (Saint-Luc-UCLouvain), Belgium.

### Participants

For the recruitment, participants were contacted by emails and phone calls based on previously existing contact lists. Thirty-three children with bilateral cerebral palsy (BCP) and 9 adults with chronic stroke (CS) who already participated in previous HABIT-ILE camps or had manifested their interest in being informed for future HABIT-ILE interventions were contacted. Only the first seven participants who responded positively to emails or phone calls were recruited (4 children with CP and 3 adults with CS; Table 1) due to the limited number of telerehabilitation devices available at this stage. Among them, three patients (BCP3, CS1 and CS2) had already participated in a previous 2-weeks on-site HABIT-ILE camp.

**Table 1:** Participants’ clinical and demographic characteristics.

BCP	Age (years)	Gender	MACS	GMFCS	More affected side	GMFM-66 (% logit)	
1	5	F	II	II	R	66	
2	6	M	III	IV	L	27	
3	10	F	III	III	L	45	
4	10	M	III	IV	R	41	
Chronic stroke	**Age (years)**	**Gender**	**Affected side**	**FAC**	**Time post stroke (months)**	**FMA-UE (/66)**	**FMA-LE (/34)**
1	63	M	L	IV	64	29	26
2	74	M	L	V	67	55	33
3	62	M	R	IV	29	55	25

BCP = bilateral cerebral palsy; F = female; M = male; MACS = Manual Ability Classification System; GMFCS = Gross Motor Function Classification System; L = Left; R = Right; GMFM-66 = Gross Motor Function Measure-66; FAC = Functional Ambulation Classification; FMA UE/LE = Fugl-Meyer Assessment for Upper Extremity/Lower Extremity.

To be included in the pilot study, children had to be diagnosed with bilateral CP and aged between 5 and 18 years. Patients with stroke were included if they suffered from an ischemic or hemorrhagic stroke for more than 6 months and were between 18 and 90 years old. A common inclusion criterion was the availability of a caregiver for 3h a day to perform the one-week telerehabilitation program (5 days). For logistical reasons (telerehabilitation device deposit), all subjects had to live within a maximum of 1 hour drive from the device manufacture. Exclusion criteria were uncontrolled seizure, botulinum-toxin injections/orthopedic surgery 6 months before the study or during the experimentation, and severe cognitive disorders limiting the understanding of simple games.

The Gross Motor Function Measure-66 (GMFM-66) [36] was used to characterize the gross motor skills of CP children so that the functional goals could be set as accurately as possible. For adults with stroke, we used the Fugl-Meyer Assessment for Upper/ Lower extremity (FMA-UE/LE) [37].

### HABIT-ILE@home intervention

HABIT-ILE is an intensive bimanual therapy requiring a constant and simultaneous implication of the trunk and lower extremities [17]. It is a goal-oriented approach, with a progressive increase in motor difficulty aiming to train movements that will help to achieve functional goals. In this study, two functional goals were defined by the patient and his/her caregiver, in agreement with the therapeutic team, during an interview at the pre-therapy assessment (see secondary outcomes: clinical measures). Each goal was first analyzed by the therapeutic team to identify movements and the different steps needed to achieve the activity. Then, the underlying motor abilities were trained according to the motor skill learning principles of HABIT-ILE: goal-oriented, fun, “hands-off” (movements are induced by modeling the therapeutic environment rather than manually guided), shaping of the difficulty, intensity of practice (voluntary active movements during most of the therapeutic time), and motivation (feedback, support, reward, fun…) to finally train functional goals. More details about HABIT-ILE principles and the way to progressively grade activities and tasks toward more complex bimanual coordination and increasing demands of the lower extremities and postural control are available elsewhere [17].

In this pilot study, HABIT-ILE was carried out by a caregiver (child’s parent, older sibling, patient’s spouse) at home, 3 hours per day (30min under supervision - 2h30 autonomously - 30min under supervision), 5 days a week. The intervention was conducted for one week, resulting in 15 hours of therapy added to their usual care. Each daily session began and ended with a 30 minutes videocall with a remote supervisor (1 out of 3h with the virtual presence of an online trained supervisor). The two daily 30-minute video calls were an integral part of therapeutic time as participants were practicing their movements in supervised activities, while receiving direct feedback on the performed movements. The role of the supervisor was multiple and crucial. First, the supervisor had to plan the content of therapeutic sessions, adapt the therapeutic plan, ensure graded difficulty during therapeutic tasks, and monitor the delivery of the intervention. Second, as patients’ caregivers/relatives were not health professionals, the supervisor had to provide remote coaching about several aspects: observation of movement skills, detection of movement compensation, adaptation of therapeutic environment, and using positive reinforcement strategies.

The first call of the day was used to start the intervention and explain the therapeutic plan of the day, with coaching about how to perform the different activities and train specific motor abilities (i.e., REAtouch Lite settings, trained movements, grasping types, objects and games to use, way to present the objects, patient positionning, duration of each activity/game, type of instructions and feedback to provide, way to adapt therapeutic environment). Between the two 30-minute calls, the participants and caregivers performed the suggested therapeutic activities based on HABIT-ILE principles in autonomy, with games and activities including the telerehabilitation device or the practice of functional activities. Each daily session ended with the second 30-minute call, allowing to observe patients performing trained tasks, check on therapeutic plan completion and, if required, correct and redirect the caregivers, answer caregivers’ questions, and gauge whether and how to increase the difficulty of motor activities for the next day. At the end of each therapy day, the supervisor sent the caregiver a therapeutic program for the next day. In addition to the daily supervision, a 30-minute training was provided the week before the beginning of the program and a “caregivers’ guide” was created to help them to perform the HABIT-ILE intervention. These included 1) explanations about motor skill learning principles used in HABIT-ILE and their application during intervention, 2) advices about how the caregiver should provide feedback to correct and motivate the patient, and 3) ways to fill in the therapy follow-up sheet to monitor each activity/game performed by the patient during the daily 3 hours.

### Telerehabilitation device

The REAtouch Lite (Axinesis S.A.) is a virtual device equipped with a touchscreen interacting with real objects manipulation and a telecommunication system allowing to remotely interact with supervisors. The telecommunication system required a stable internet connection (Wi-Fi or cabled) and included an individual Microsoft Teams account and a rotative camera (Logitech PTZ Pro) with a zoom and a remote control by supervisors (for a better visibility of the participants’ environment).

REAtouch Lite uses the same software as the REAtouch, a previous less transportable device. It includes games (same for children and adults) that can be set to be played uni- or bimanually, with small or large objects according to therapeutic needs. A recent study [38] showed significant improvements in motor functions of the upper extremities, daily activities, and functional goals attainment in children with unilateral CP while using the REAtouch half the time during a HABIT-ILE camp. The smaller size of the REAtouch Lite makes it feasible to be used at the patients’ home.

**Fig 1:**
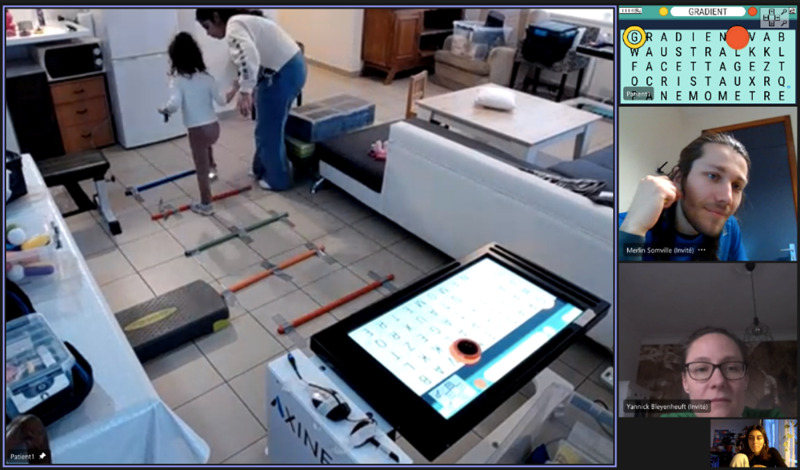
Illustration of the HABIT-ILE@home intervention with the use of the REAtouch Lite and the therapeutic environment in a remote supervision.

In addition to the telerehabilitation device, additional materials were delivered to enrich the therapeutic environment. It was composed of boxes with specific objects to manipulate, adaptation material (tape, anti-slippery tissue) and sitting/standing furniture (adaptable bench, swissball, steps) (Fig 1).

### Outcomes

#### Feasibility.

Two questionnaires (for CP or CS subjects) were used at the post-intervention testing session to measure the feasibility of a HABIT-ILE telerehabilitation program, its acceptability and the patients’ and caregivers’ compliance with treatment. These feasibility questionnaires were developed based on seven existing questionnaires: System Usability Scale [39] (measuring the ease of use of an interactive system), User Experience Questionnaire [40] (collecting users’ subjective feeling of their experience with technological products), AttrakDiff2 [41,42] (describing users’ experience with interactive systems), Dimensions of Mastery Questionnaire [43] (measuring subjects’ motivation and perseverance in mastering a challenging skill or task), Intrinsic Motivation Inventory [44] (assessing subjects’ intrinsic motivation), Exercise Adherence Rating Scale [45] (measuring subjects’ adherence to prescribed home exercises), and the Exercise Therapy Burden Questionnaire [46] (assessing the burden of exercise therapy).

The questionnaire dedicated to children with CP contained 71 items. For 61 of them, caregivers were required to give their degree of agreement on a 4-levels scale ranging from “disagree” (0) to “agree” (3). More precisely, the 61 items investigated four important dimensions related to the telerehabilitation device and the remote therapy: the REAtouch Lite interactive system features (5 subdimensions, 21 items), the perceived application of HABIT-ILE therapy in the home setting (3 subdimensions, 16 items), the caregivers’ compliance (5 subdimensions, 16 items), and the patients’ motivation/involvement (2 subdimensions, 8 items). The 10 remaining items were answered by children themselves to investigate their compliance with treatment through 4 dimensions: REAtouch Lite related questions (3 items), children’s enjoyment (3 items), children’s perceived burden of the therapy (2 items), and children’s engagement (2 items). The scoring of these items was adapted in a dichotomous way (on a 4-levels scale but the question was asked in two steps) for a better understanding by the children.

The questionnaire dedicated to adults with CS included 72 items. The same four important dimensions related to the telerehabilitation device and the remote therapy were assessed through 62 items (one supplementary item for the dimension related to the device). REAtouch Lite interactive system features were assessed by patients themselves while caregivers’ compliance was evaluated by caregivers. HABIT-ILE@home therapy was appreciated either by caregivers alone (9 items) or in agreement with patients (7 items). Patients’ motivation/involvement was assessed either by patients (6 items) or by caregivers (2 items). Finally, the 10 remaining items were only answered by patients to investigate their compliance with treatment through 3 dimensions: patients’ perceived usefulness of HABIT-ILE@home (2 items), patients’ enjoyment (4 items) and patients’ perceived burden of the therapy (4 items). The degree of agreement was measured on the same 4-levels scale as the previous one.

The submission of the feasibility questionnaires was performed through interviews allowing also qualitative data collection.

#### Functional goals.

For both children with CP and adults with CS, the Canadian Occupational Performance Measure (COPM) [47] was used to define the two functional goals that were trained during HABIT-ILE@home and to assess the patients’ performance in achieving them (“efficiency”) as well as the patients’ and caregivers’ satisfaction with the performance achieved before and after the intervention on a response scale from 1 to 10.

### Data analysis

All data are available in S1 File. Feasibility data are presented in radar charts using Sigmaplot 14.5. For each subdimension of the questionnaire, an average score was first calculated from the responses of each participant through the different items of the subdimension. Then, the median value of the different individual mean scores was computed within each dimension. These quantitative results were enriched by qualitative information from patients/caregivers’ feedback. No statistical analysis was conducted to compare COPM scores before (T0) and after (T1) HABIT-ILE@home due to the small sample size in each subgroup. Instead, individual observations about COPM score changes (T1-T0) regarding the minimal clinically important difference (MCID: 2 points) [48] were provided.

## Results

### Feasibility

**Fig 2:**
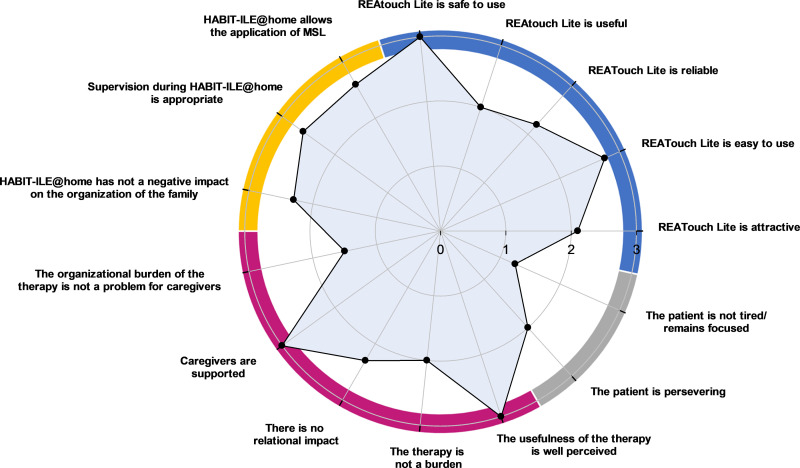
Agreement of caregivers (n ** = 4) of children with bilateral cerebral palsy** (0 = disagree, 1 = somewhat disagree, 2 = somewhat agree, 3 = agree). Four dimensions related to the telerehabilitation device and the remote therapy are investigated: REAtouch Lite features (upper right dial, blue), the therapy itself (upper left dial, yellow), caregivers’ compliance (lower left dial, purple) and patients’ motivation/involvement (lower right dial, grey). Dots indicate the median values of caregivers’ responses in each subdimension. MSL: motor skill learning.

**Fig 3:**
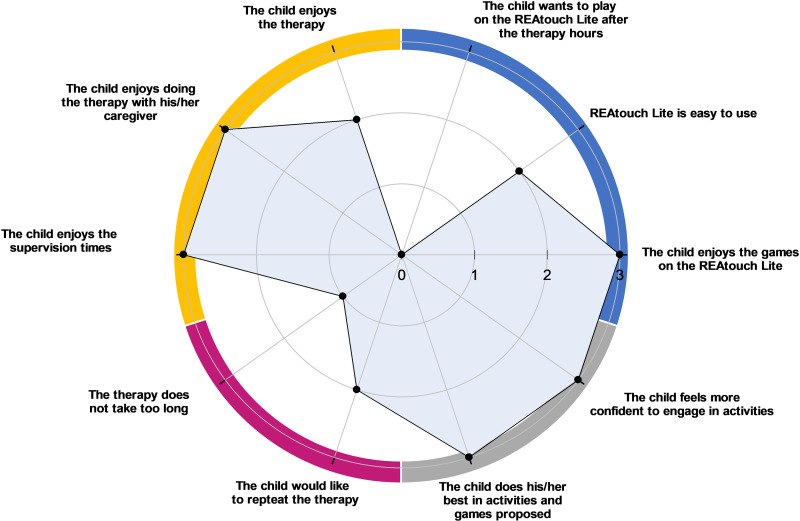
Agreement of children with cerebral palsy (n ** = 3) about their compliance with treatment and their enjoyment** (0 = disagree, 1 = somewhat disagree, 2 = somewhat agree, 3 = agree). Four dimensions were investigated: REAtouch Lite related questions (upper right dial, blue), children’s enjoyment (upper left dial, orange), children’s perceived burden of the therapy (lower left dial, purple) and children’s engagement (lower right dial, grey). Dots indicate the median values of children’s responses in each subdimension.

**Fig 4:**
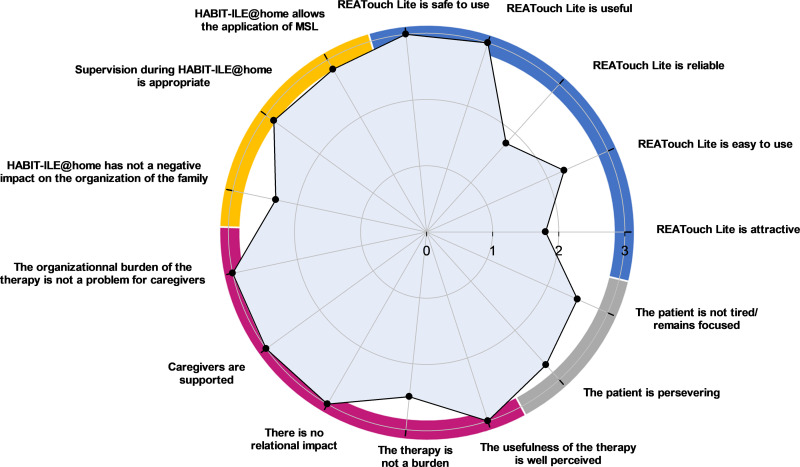
Agreement of adults with chronic stroke and their caregivers (n ** = 3) (0 = disagree, 1 = somewhat disagree, 2 = somewhat agree, 3 = agree)**. Four dimensions related to the telerehabilitation device and the remote therapy are investigated: REAtouch Lite features (upper right dial, blue), the therapy itself (upper left dial, yellow), caregivers’ compliance (lower left dial, purple) and patients’ motivation/involvement (lower right dial, grey). Dots indicate the median values of patients’ and caregivers’ responses in each subdimension. MSL: motor skill learning.

**Fig 5:**
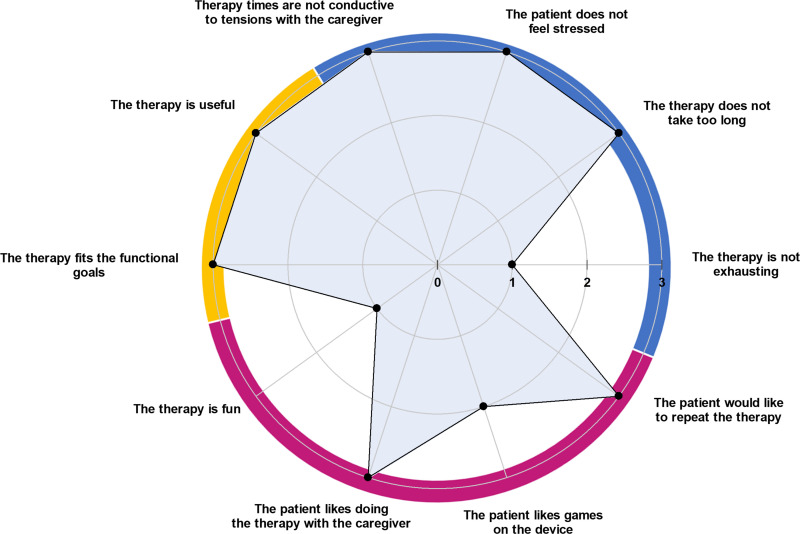
Agreement of adults with chronic stroke (n ** = 3) about their compliance with the treatment and their enjoyment (0 = disagree, 1 = somewhat disagree, 2 = somewhat agree, 3 = agree).** Three dimensions were investigated: patients’ perceived usefulness of HABIT-ILE@home (upper left dial, yellow), patients’ burden of the therapy (upper right dial, blue) and patients’ enjoyment (lower dial, purple). Dots indicate the median values of patients’ responses in each subdimension.

The quantitative results of feasibility questionnaires are shown in Fig 2 and Fig 3 for children with bilateral CP and in Fig 4 and Fig 5 for adults with CS.

#### Interactive system features: REAtouch lite.

Overall, respondents reported a good experience with the REAtouch Lite. It was considered as safe due to its stability (CP and CS medians = 3), easy to use (CP median = 2.8; CS median = 2.3) and useful (CP median = 2; CS median = 3). Although learning to use the device was considered as easy, the interface and browsing were less intuitive for adult patients. All subjects reported that the REAtouch Lite was an added value to the therapy thanks to its playfulness, without being a key element. Attractiveness and reliability were scored higher for children with CP (medians = 2.1 and 2.2, respectively) than for stroke patients (both medians = 1.8).

#### Therapy@home.

Caregivers reported a self-perceived overall ability to apply motor skill learning therapeutic principles during home training (applying a hands-off, structured and intensive intervention, promoting motivation and giving feedback, training functional goals) (CP median = 2.6; CS median = 2.8). Nevertheless, children’s caregivers reported difficulties to maintain the child motorically engaged throughout the therapy (item: “It was easy to keep the child motorically active” median = 1). The therapeutic supervision was perceived as adequate for both samples (CP median = 2.6; CS median = 2.9). The impact of HABIT-ILE@home was relatively positive on family organization and perceived as comfortable and timesaving (CP median = 2.3; CS median = 2.7) but the therapy timetable (item: “Therapy doesn’t take too long”) has been reported as very constraining for children’s parents (median = 1) and the CS patient’s spouse in employment (score of 1). In contrast, it was not a problem for patients’ other retired spouses (median = 3). Note that it was very complicated, for one caregiver (BCP2), to apply HABIT-ILE@home due to severe cognitive impairment of the child limiting the possibilities of interaction and maintained attention with the virtual environment.

#### Caregivers’ adherence.

The impact of the therapy on daily organization was considered as heavy for children’s caregivers (median = 1.5). However, adults’ caregivers did not report such an impact (median = 3). Caregivers of both samples felt fully supported by the supervisors and their surroundings (both medians = 3), perceived very well the usefulness of the therapy (both medians = 3), and reported no major impact of the therapy on their relationship with their child or spouse (CP median = 2.3; CS median = 3). The burden of the therapy was reasonable for the caregivers (CP median = 2; CS median = 2.5) but all of them found their role as therapist exhausting (item: “my role as a caregiver was not exhausting”, both medians = 0).

#### Patients’ motivation and involvement in the therapy.

Perseverance in the therapeutical activities was slightly higher in CS (patients’ and caregivers’ point of view, median = 2.7) than in CP (caregivers’ point of view, median = 2.0). According to children’s caregivers feedback, that would be due to their young age and their need to switch to another activity when they got bored or faced difficulties. Stroke patients were not distracted by their environment and the fatigue caused by the therapy did not prevent them from pursuing the therapy (median = 2.5). On the opposite, the environmental distraction and the fatigue caused by the therapy could be obstacles to the children’s motivation and involvement in the therapy (median = 1.3).

#### Compliance with treatment and enjoyment.

As illustrated in Fig 5, post-stroke patients did not find the therapy fun (median = 1) but would like to repeat it (median = 3) because of its extreme usefulness (median = 3). According to CS adults, the only significant reported burden of the therapy was tiredness (median = 1).

Three out of the 4 children could respond to the ten items about their compliance with the treatment and their enjoyment as one child had cognitive disorders. As shown in Fig 3, children with CP enjoyed the therapy (median = 2), especially with the virtual games of the device (median = 3), but all of them perceived it as too time consuming (median = 1) and they did not want to play with the device after the therapy hours (median = 0). Although all children felt more confident in getting engaged in relevant functional tasks (median = 3), only two of the three children would like to get this therapy again (median = 2).

### Functional goals

**Fig 6:**
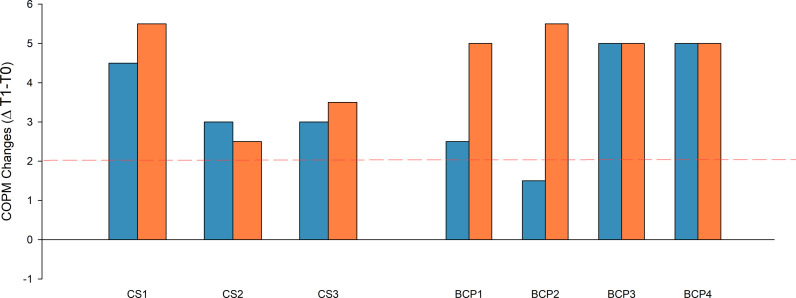
Score changes of the Canadian Occupational Performance Measure (COPM) before (T0) and after (T1) HABIT-ILE@home therapy for both chronic stroke patients (left, CS) and children with bilateral cerebral palsy (right, BCP). Blue bars present changes in performance and orange bars present changes in satisfaction. Changes are calculated based on the difference (Δ T1-T0) of the mean scores of the two functional goals (set at baseline). The dotted red line presents the COPM minimal clinically important difference (MCID: 2 points).

COPM score changes (T1-T0) for both performance and satisfaction regarding functional goals are presented in Fig 6. Every participant improved over the minimal clinically important difference (MCID) for satisfaction while everyone except BCP2 improved over the MCID for performance.

## Discussion

Previous home-based interventions for children with cerebral palsy (CP) have been explored [49], and there is a growing interest in telerehabilitation for stroke patients in the chronic phase [50]. However, the implementation of evidence-based therapies in a remote setting remains challenging. It is crucial to investigate the factors that facilitate or hinder the successful delivery of these interventions in a home environment.

This pilot study aimed to assess the feasibility of HABIT-ILE@home delivered by caregivers in the home setting with remote supervision from a therapeutic team which may provide a program still requiring therapeutic supervision, but without the need of a one-to-one therapist for each patient. Caregivers reported feeling adequately supported by the supervision team to carry out HABIT-ILE@home sessions thanks to appropriate clinical supervision. Moreover, HABIT-ILE principles seemed to be transferable at patients’ home. Although the motivational source differed between children (the therapy is fun) and adults (the therapy is useful), both children with CP and adults with stroke as well as their caregivers presented an overall good adherence to treatment. However, some specificities between CP and stroke patients in terms of feasibility and compliance were identified for each population and will be discussed. Despite the small dosage provided in this study, meaningful improvements in the performance and the satisfaction of functional goals were shown.

### Interactive system

HABIT-ILE@home has been supported by an adequate telerehabilitation device (REAtouch Lite) according to the perceptions of both CP and CS participants. The interactive system seems helpful to provide remote supervision, and to set up therapeutic activities. As initially developed to provide therapists with opportunities to apply a motor skill learning based intervention such as HABIT-ILE [38], the REAtouch Lite seemed appropriate to accompany caregivers throughout the application of the HABIT-ILE@home intervention. Nevertheless, CS patients are less attracted by the device and the implemented games than children and felt more disturbed by technological bugs and telecommunication issues. Actually, some participants reported occasional device reliability problems (mainly games that shut down, touchscreen bugs). This will need to be fixed and considered in the future.

### Therapy@home and supervision

In 2017, Ferre et al. emphasized the importance of the supervision in the context of home-based HABIT (H-HABIT) as bimanual gains were observed when the supervision was conducted in-person (not remotely) during their feasibility study [51]. However, they did not observe such results while a weekly remote supervision for 10% of therapy time through calls was used in their RCT. Therefore, in our feasibility study, we tried to apply a more frequent remote supervision as close as possible to face-to-face. The aim was to ensure sufficient therapeutic guidance/coaching of caregivers and secure a proper application of the treatment plan. Moreover, seeing patients twice a day allowed to precisely shape the difficulty of therapeutic activities (objects manipulations, trunk control solicitation, spatial/temporal constraints…) which is a key HABIT-ILE component [17]. Likewise, close supervision with frequent interactions with patients and caregivers allows to provide precise daily plannings of games and activities (based on patients’ preferences). This closer supervision may have prevented decrease in intensity and ensured adequate motor engagement time. Supervision sessions were also useful to maintain motivation along therapy hours (encouragements, positive rewards, …) and to solve technical problems (use of the device or telecommunication problems). Both CS and CP caregivers were satisfied with the remote supervision and reported confidence in applying motor skill learning principles during intervention. Nevertheless, CP caregivers reported difficulties maintaining children active during the entire therapy time (median = 1) in contrast to CS caregivers (median = 3), meaning that a closer support in terms of motivation and daily organization should probably be considered to ensure a high intensity in children. It could include shorter but more diversified games and activities taking children’s preferences into account.

### Caregivers’ adherence

In previous studies [38], the crucial role of therapists during virtual-based interventions was highlighted. In a remote setting, caregivers are the keystone between the therapeutic supervisor and patients as they implement the proposed activities and therapeutic elements at home (shape and adapt the therapeutic environment, provide feedback on motor performance and positive reinforcement, provide safety, etc). Caregivers reported a higher organizational burden in children than in CS group. This is likely explained by the younger age of children’s caregivers potentially implying to deal with professional and family requirements (householding, other children…) in their organization. This point highlights the need for caregivers to be fully engaged in the therapy during dedicated specific times. It includes personal time management and planning flexibility from the supervision team for such therapeutic modalities.

No major impact of the therapy was observed on the patients-caregivers relationship in our pilot study. Although both children with CP and stroke adults enjoyed doing the therapy with their caregivers, The “therapeutic role” was felt to be slightly more burdensome for children’s caregivers than for adults’ ones. It may be explained by parental authority that could have been somehow modified due to the principles of the therapy while children experienced difficulties (e.g., always positive feedback, intensity, non-guided voluntary movements…). The absence of significant emotional burden experienced by caregivers in their dual role as both relatives and therapists need to be confirmed in studies with larger sample sizes, especially in children with CP.

### Patients’ compliance with treatment and enjoyment

Motivation is a crucial aspect to ensure the patient’s engagement in an activity [52] and maintaining it over the long term in rehabilitation using virtual devices is a major challenge [53]. According to the Self-Determination Theory (SDT), the autonomous motivation is defined as engaging in a behavior because it is perceived as consistent with relevant goals or outcomes and emanates from the self in contrast to an extrinsic controlled motivation, based on pressure, demand or obligation [54]. This autonomous motivation can be based on an intrinsic or internalized extrinsic motivation. Interestingly, our results showed good adherence to treatment in both children and adults. Children’s motivation would rather be related to an internalized extrinsic motivation (play activities during interventions and positive reinforcement mechanisms) while adults would rather refer to purely intrinsic motivation based on the expectation of achieving their functional goals. Still acting as an internalized motivation, the implementation of a virtual device in rehabilitation may often be considered as enhancing children involvement in their rehabilitation [55]. Although the motivation of adults does not seem related to games, offering playful activities for adult patients may still facilitate long-term motivation, especially considering higher dosage remote interventions. Finally, the presence of a caregiver with the patient during the intervention should have an impact on adherence. In fact, previous research highlighted the importance of human connections for compliance with treatment [56].

In this pilot study, we included patients with various levels of motor and cognitive impairments to highlight the potential to manage the therapy process at home with associated symptoms.

This led to one participant (BCP 2) with cognitive impairments having trouble following the process as initially presented due to a lack of interest in virtual games and difficulties in interacting with the supervision team. This observation suggests that therapy at home for patients with such profile should be considered with adaptations, notably activities meaningful for the patient, presumably not within the REAtouch Lite environment.

In previous home-based virtual reality programs, fatigue is known to be responsible for a lower compliance/motivation [57,58]. In this pilot study, fatigue has been reported for all patients. In addition to the 3 hours of therapy planned every day, many of the participants/caregivers carried out their usual activities such as school, work, family activities, etc. This might be solved by specific time allocated to therapy exclusively by the caregiver or alternately by proposing such interventions with a lower dosage By coaching caregivers and giving clear instructions regarding breaks or type of games and their duration, we might be able to deal with participants’ fatigue along the therapy (alternate different types of activities with high and low energy expenditure, with or without the virtual device, while maintaining a high motor engagement time, etc.).

### Functional goals

In this study we observed changes in the perceived performance in functional goals and the associated satisfaction. These results are consistent with Jackman et al. [59] who have suggested a minimal threshold of 14–25 therapy hours to observe improvements in functional goals for upper extremities in children with BCP. However, a larger amount of therapeutic time would be necessary to expect clinically relevant improvements in motor and functional abilities [59].

### Limitations

This study has several limitations. First, as a pilot study with a small sample size, its primary focus was on testing the experimental design and feasibility of HABIT-ILE@home rather than establishing its efficacy. Consequently, future studies with randomized controlled designs and larger sample size are needed to investigate the non-inferiority of this new modality regarding the on-site HABIT-ILE intervention. In addition, three participants (CS1, CS2 and BCP3) had already experienced a HABIT-ILE camp before this study. It may have influenced satisfaction and adherence to the study. However, considering the therapy modality (e.g., use of the virtual device, coached relatives/caregivers interventionists instead of therapists, lack of group effect), the experience of the previous camp should have limited impact on the results (see S1 File).

Second, the therapeutic content’s completion was documented through subjective reports from patients and caregivers. The present study lacks objective measurements to monitor the time spent using the REAtouch Lite device or engaging in functional activities (manual [bimanual vs. unimanual], postural [sitting or standing] or locomotor tasks) as well as specific objective tools to fully characterize the participants, especially regarding cognitive impairments. Future research should include objective tools or methods to document therapeutic content.

Finally, satisfaction and performance regarding functional goals were evaluated through subjective scoring, which may be influenced by caregivers’ and participants’ expectations or desires for improvement.

## Conclusion

To conclude, this pilot study shows that HABIT-ILE carried out by a patient’s caregiver seems to be reasonably transposable at patients’ home with an adapted framework and adequate supervision. Overall, very good results were observed regarding the application of motor skill learning principles at home and adherence to the treatment seems to be guaranteed, leading to improvements in functional goals achievement. However, fatigue and organizational burden of the therapy suggest that there are some fields of improvement to this adaptation of the therapy, even more if we intend to reach the same dosage as previously tested during on site HABIT-ILE camps (5-9h/2 weeks). This study provides good indications and suggestions for further randomized controlled studies with larger samples of patients and a higher amount of therapy hours with the aim to establish the efficacy of HABIT-ILE@home.

## Supporting information

S1 File Feasibility questionnaire_CS. Data for the feasibility questionnaire in patients with chronic stroke. **Feasibility questionnaire_BCP.** Data for the feasibility questionnaire in children with bilateral cerebral palsy. **COPM.** Canadian Occupational Performance Measure (COPM) data for patients with chronic stroke and children with bilateral cerebral palsy.
(XLSX)
